# Characteristics of Yoga Providers and Their Sessions and Attendees in the UK: A Cross-Sectional Survey

**DOI:** 10.3390/ijerph19042212

**Published:** 2022-02-15

**Authors:** Gamze Nalbant, Sarah Lewis, Kaushik Chattopadhyay

**Affiliations:** 1 Lifespan and Population Health Academic Unit, School of Medicine, University of Nottingham, Nottingham NG5 1PB, UK; sarah.lewis@nottingham.ac.uk (S.L.); kaushik.chattopadhyay@nottingham.ac.uk (K.C.); 2 The Nottingham Centre for Evidence-Based Healthcare: A JBI Centre of Excellence, Nottingham NG5 1PB, UK; 3 Clinical Sciences Building B126, University of Nottingham, Nottingham NG5 1PB, UK

**Keywords:** yoga, cross-sectional survey, UK

## Abstract

Yoga is an ancient Indian philosophy and way of life that is being used as a method of improving health and wellbeing. Evidence shows that yoga has several health benefits, such as managing many noncommunicable diseases, such as hypertension, and improving mental health. The popularity of yoga is growing in the UK, but it is mostly unregulated with little information available about yoga providers and their sessions and attendees. This study aimed to explore who is providing yoga; what sessions are available, where, and at what cost; and who attends these sessions in the UK and whether yoga providers were aware of health conditions in their sessions. A cross-sectional survey was undertaken among yoga providers in the UK. They were approached through four major UK yoga associations. In total, 407 yoga providers participated. Most providers were aged 45–64 years (69%), female (93%), and white (93%). The median number of group sessions and one-to-one sessions delivered per week was four and two, respectively. The most common styles were Hatha (28%), Iyengar (26%), and Vinyasa (15%). Sessions had a varying emphasis on different yogic practices, but 59% of providers allocated most time to yogic poses (asana), 18% to breathing practices (pranayama), and 12% to meditation (dhyana) and relaxation practices. Most (73%) reported that their attendees disclosed their health conditions to them, most commonly mental health issues (41%), hypertension (25%), and heart diseases (9%). This study showed that yoga sessions are widely available in the UK, often provided and practiced by women, and concentrate on yogic poses. Sessions concentrate on the asana and tend not to include many of the more holistic aspects of yoga that are practiced in South Asian countries. Yoga providers are often aware of health conditions but may benefit from training to deliver sessions suitable for specific health conditions.

## 1. Background

Yoga, an ancient system of health and way of life, originated in the Indian subcontinent over 5000 years ago [1]. There are different branches of yoga, each with a different emphasis on and approach to practice. Of the six main branches of yoga, Hatha yoga is the most commonly practiced style, and it is an overarching term for physically based yoga styles, such as Iyengar, Asthanga, and Kundalini [2,3]. Yoga, as defined by Patanjali (the author of the textbook of classical yoga), has eight components, which are ethical standards (yama), self-discipline (niyama), yogic poses (asana), breathing practices (pranayama), withdrawal of the senses (pratyahara), concentration (dharana), meditation (dhyana), and transcendence (samadhi) [4,5].

Yoga helps discipline the body and mind through low- to moderate-intensity physical activities, breathing practices, meditation and relaxation practices, and a healthy lifestyle [6]. It requires no or very little equipment and can be practiced indoors and outdoors [6]. It is usually safe even in individuals with comorbidities [6,7,8]. There is growing evidence to support the use of yoga as a method of improving health and wellbeing. Numerous systematic reviews have shown that yoga appears to improve a range of health conditions, including hypertension, mental health issues, heart diseases, musculoskeletal conditions, respiratory diseases, diabetes, obesity, and stroke [9,10,11,12,13]. 

Yoga has a high acceptance rate in South Asian countries, such as India and Nepal, and it is widely practiced in these countries [14,15]. The Ministry of Ayush in India is dedicated exclusively towards traditional therapies, including yoga, and yoga is part of the Indian health care system [16]. In terms of yoga education, there are bachelor’s degree, which is a recognized medical course in India [17], and postgraduate teaching and research degrees [18]. Higher education and practice is not currently regulated in India as it should have been, but there are some initiatives to regulate these. The Indian government initiated the Scheme for Voluntary Certification of Yoga Professionals, which is monitored by the Ministry of Ayush [19,20], and is creating a regulatory body for higher education and practice [21,22]. Similarly, Nepal has also established yoga as a health promotional tool for noncommunicable disease prevention and control [23]. Yoga is also popular in other parts of the world, including several high-income countries, such as Australia, Germany, and the U.S. [24,25,26,27].

Yoga is also growing in popularity in the UK [28,29]. A representative study of 1106 people in the UK found around 10% of participants practiced yoga, and around 13% of participants practiced meditation [29]. In the UK, there are approximately 10,000 yoga teachers [24] and a further 150 yoga therapists, that is, yoga teachers with additional training and experience in therapeutic adaptation and application of yoga to those with health issues [30], and they do not have to be recognized health professionals in the UK. The UK’s National Health Service (NHS) suggests yoga as a safe and beneficial practice for people with a variety of health conditions [7], and NHS provides yoga sessions for the staff in some places [31,32,33]. Despite the popularity of yoga and its potential health benefits, yoga does not feature as a health and well-being approach for patients within the NHS [32]. Although there are yoga associations in the UK providing guidance and setting requirements for their members, registration with these organizations is entirely voluntary. Yoga is unregulated in the UK, and there are few data about yoga practice [34]. If yoga is to be recommended for those with health conditions in the UK, it is important to understand what is being provided and by whom, who attends yoga sessions, how accessible it is across the country, and whether yoga providers know anything about the health conditions of their attendees. Therefore, this study aimed to find out who is providing yoga; what types of yoga sessions are available, where, and at what cost; and who attends these sessions and whether yoga providers were aware of health conditions in their sessions. 

## 2. Methods

### 2.1. Study Design

We conducted a cross-sectional study of yoga providers using an online survey and a descriptive approach.

### 2.2. Study Participants and Eligibility Criteria

Yoga providers (i.e., teachers and therapists) were eligible if they were at least 18 years old and delivered a yoga session at least once in a fortnight during the last six months in the UK prior to the survey date.

### 2.3. Data Collection Tool

A web-based survey questionnaire was developed and piloted among yoga providers (three yoga teachers and one yoga therapist). The questionnaire was administered through Jisc Online Surveys, formerly known as Bristol Online Surveys software [35]. The questionnaire included 32 questions and collected data on (i) the sociodemographic characteristics of yoga providers and their yoga training; (ii) the delivery of yoga sessions; (iii) the styles of yoga taught and the components practiced in a yoga session and (iv) the awareness of yoga providers of health conditions, including hypertension, diabetes, obesity, heart diseases, stroke, chronic kidney disease, respiratory diseases, and mental issues. As this survey was conducted as part of a PhD looking at yoga for hypertension, we also asked a specific question on whether yoga providers had received training on hypertension and if they required further training.

### 2.4. Recruitment and Study Period

Yoga providers were approached through professional yoga associations in the UK. Five yoga associations were approached to circulate the survey among their members, and four agreed, namely Association for Iyengar Yoga in the UK and Republic of Ireland, British Wheel of Yoga (BWY), Complementary and Natural Health Council (CNHC), and Independent Yoga Network. They either emailed their members an invitation letter with the survey link or posted the study details on their website and Facebook page. Data collection took place from 3 March to 3 September 2020. The participant information sheet was provided at the beginning of the study, and those interested gave written informed consent. Participation in this study was entirely voluntary. Participants were entered into a prize draw of GBP 100 if they provided their email addresses. Participation in the prize draw was entirely voluntary, and confidentiality was protected in accordance with regulatory requirements. Email data were not downloaded, and participants were not identifiable in any process.

### 2.5. Sample Size

The required sample size was estimated on the basis of the accuracy of estimates of proportions, for example, of the proportion delivering a certain style of yoga. A sample size of 384 was sufficient to estimate proportions to within 5% error using a 95% confidence interval. A larger sample was approached presuming that not all would respond.

### 2.6. Data Analysis

Mean and standard deviation (SD) were calculated for normally distributed continuous variables, median and interquartile range (IQR) for skewed continuous variables, and number and percentage for categorical variables. Responses of yoga teachers and therapists were compared using the Wilcoxon Rank Sum test for continuous variables and the chi-square test for categorical variables. If more than 20% of cells had a value <5, Fisher’s exact test was preferred over the chi-square test. Data were analyzed using Stata16 (StataCorp, College Station, TX, USA) [36]. Regions of the UK were based on the Office for National Statistics (ONS) 2017 data and were divided into 12 regions: Wales, Scotland, Northern Ireland, North East, North West, Yorkshire, and the Humber, East Midlands, West Midlands, East, London, South East, and South West [37]. Missing data were reported for all variables but were not included in the analysis. The level of statistical significance was set at *p* ≤ 0.05. 

## 3. Results

### 3.1. Socio-Demographic Characteristics of Participants

The socio-demographic characteristics of participants are presented in Table 1. A total of 407 yoga providers (i.e., 340 yoga teachers and 67 yoga therapists) from all the regions of the UK completed the survey. They were mostly aged 45–64 years (69%), female (93%), and white (93%). They were mostly self-employed (82%) and had a diploma degree in yoga (46%). Only around 8% of them stated that they were a member of a regulated healthcare profession. Yoga therapists tended to have a higher rate of yoga educational level than yoga teachers. In addition, they had a significantly higher rate of self-employment and longer years of delivering yoga sessions compared to yoga teachers. 

### 3.2. Structure, Delivery, and Cost of Yoga Sessions

Yoga sessions were available as group and one-to-one sessions though the majority of participants (95%) delivered group sessions (Table 2). The delivery of one-to-one sessions was more common among yoga therapists (66%) than yoga teachers (24%). The median number of group sessions and one-to-one sessions delivered per week was four (IQR = 2, 6) and two (IQR = 1, 3), respectively, and the group size was 10 (IQR = 8, 15). Yoga therapists delivered more group and one-to-one sessions than yoga teachers in a week, and interestingly, the number of attendees in a group session was higher for yoga therapists. Eighty percent of participants were providing evening sessions. Many also delivered sessions at the weekend (25%) though weekend sessions were more common for yoga therapists (40%) than yoga teachers (22%). 

Sessions were available in all the ONS regions of the UK although South West (14%), South East (13%), and London (13%) were the most common regions among respondents (Table 2). Sessions were available in private, public, and public and private partnership settings but mostly delivered in private settings (60%). The median cost per group session was GBP 7 and per one-to-one session was GBP 40, and the cost was higher in the sessions delivered by yoga therapists. The cost of the sessions was mostly paid out-of-pocket by attendees (82%), and a very small fraction was public-funded (6%). The majority of the participants (63%) reported that 75–100% of the attendees in a session were female.

### 3.3. Style and Content of Yoga Sessions

Participants reported using different styles of yoga, but the most common styles reported were Hatha (28%), Iyengar (26%), and Vinyasa (15%) yoga (Table 3). Hatha yoga was more common among yoga teachers (30%), and Iyengar yoga was more common among yoga therapists (28%). Over half of the participants (68%) used only one style of yoga in their sessions. Sessions had a varying emphasis on different yogic practices, but 59% of providers allocated most time to asana, 18% to pranayama, and 12% to dhyana and relaxation practices. Yoga therapists gave a slightly higher emphasis on pranayama, dharana, pratyahara, niyama, yama, and samadhi. Missing data were high for the questions of frequency of yama, niyama, pratyahara, dharana, and samadhi.

### 3.4. Yoga Providers’ Awareness of Health Conditions

The majority of participants (73%) reported that attendees disclosed their health conditions (Table 4). They reported that the most frequently disclosed health condition was mental health issues (41%) and it was followed by hypertension (25%) and heart diseases (9%). When we asked specifically about training on hypertension, 87% said they had received training but 186 yoga providers, almost half of the participants, said they needed further training to deal with it and to provide a safer yoga practice. 

## 4. Discussion

We have provided a contemporary description of yoga providers and their practice across the country; we found that yoga providers were mostly aged 45–64 years, female and white, and yoga was mostly practiced by women. The predominance of women in both delivery and practice in the UK is similar to that seen in previous surveys here [34,38] and other high-income countries [26,27,39] and contrasts with findings from India where practitioners were more likely to be men [40,41]. The potential reason for the popularity of yoga among men in India might be that the roots of yoga and the growth of yoga as a practice were predominantly in males [42]. However, the findings of this study imply that yoga is perceived to be something more of interest to women than it is to men in the UK, and there may be a need to look at why that is the case. 

Yoga sessions were available in the evenings and on the weekends, which makes them accessible for those who are not able to attend sessions during day times on weekdays. Because of the wide availability of group yoga sessions and the significant difference between the cost of group and one-to-one sessions, group sessions appear to be more affordable and accessible for a wider range of people than one-to-one sessions.

Different styles of yoga were reported in this study, with Hatha and Iyengar being the most commonly delivered styles. Hatha yoga is an overarching term for physically based yoga practices, and the high rate of the report of Hatha yoga possibly suggests that Hatha yoga appears to be a common UK parlance used for any type of yoga. Our study found that yoga, as practiced in the UK, concentrates on asana and to a lesser extent pranayama and dhyana and relaxation practices, and this is similar to that seen in other high-income countries, such as Australia [43] and Germany [44]. However, this finding suggests that yoga practice in the UK tends not to include many of the more holistic aspects of yoga that are practiced in South Asian countries [45], where many of the yoga trials have been conducted. Therefore, it is important to be aware that the practice of yoga varies between contexts in terms of the styles and emphasis given to components [41]. 

This study showed that attendees commonly disclosed their health conditions, and the high rate of disclosure suggests that yoga providers were generally aware of the health conditions of their attendees. The most commonly disclosed health conditions in yoga sessions were mental health issues and hypertension, both in our study and in studies from the UK and other countries [34,38,39]. There is growing evidence on the benefits of yoga for a variety of health conditions, including mental health conditions [10,46] and hypertension [11], and yoga is commonly being used as a way for supporting well-being and managing a variety of health issues [34]. Whilst good quality studies are still needed to confirm the effectiveness of yoga in specific conditions and to determine which aspects of yoga are most beneficial, it seems likely that yoga may have a role to play in the management of a range of health conditions. If so, there may even be a role for offering yoga within the NHS. If yoga were to be more actively promoted, it is likely that sessions run by yoga teachers would be a more feasible way of providing yoga to large numbers of people because of the wider availability of yoga teachers and the cost of sessions provided by yoga teachers. Although yoga providers, including teachers, do often find out about the health conditions of their attendees, they may not have enough information on how to deliver yoga sessions to people with specific conditions, and hence, they may need some further training to deal with different conditions. When we asked specifically about hypertension, we found that most yoga providers have had some kind of training for this, but most felt they needed further training to deal with it and to provide a safer yoga practice.

### 4.1. Implications for Practice

Yoga is unregulated in the UK, and this might mean that yoga teachers with no proper training can deliver yoga sessions in the UK. People with health conditions attend these sessions; therefore, there is a need for an initiative to better regulate yoga practice in the UK. In addition, there is clearly a need for better training for yoga teachers in supporting specific health conditions, especially for mental health issues, hypertension, and heart disease, which were found to be the most commonly disclosed health conditions in this study. It is also important for yoga providers to be aware of their attendees’ health conditions to provide safer practice.

### 4.2. Implications for Research

Yoga is understudied in the UK, and our findings showed that further studies are warranted. One important question to be further investigated is the knowledge, experiences, and attitudes of yoga providers in relation to managing health conditions in their sessions and that of health professionals in recommending yoga for people with these conditions. In addition, future studies should aim to explore the motivations of the attendees and reasons for the popularity of yoga among women in the UK.

### 4.3. Strengths and Limitations

To the best of our knowledge, this was the first study describing the characteristics of yoga providers and their sessions and attendees in the UK. The standard steps in questionnaire development (design and pretesting) were followed to ensure the validity and reliability of the questionnaire. Missing data were low in this study. An online survey was conducted, so we may have missed those who have accessibility issues to online platforms. It was not possible to know the exact number of yoga providers in the UK because registration with a yoga association is not mandatory in the UK, and yoga providers can register with more than one association, so we cannot estimate accurately our response rate. However, as far as we are aware, there are around 150 yoga therapists in the UK, and we were, therefore, able to access most yoga therapists delivering sessions in the UK. The survey was conducted during the COVID-19 pandemic, an unprecedented time for everyone, which is likely to affect the delivery of yoga sessions; however, we requested participants to report their usual yoga sessions. 

## 5. Conclusions

This study shows that yoga sessions are widely available in the UK and often provided and practiced by women. Sessions concentrate on the asana and tend not to include many of the more holistic aspects of yoga that are practiced in South Asian countries. Yoga teachers are often aware of health conditions but may benefit from training to deliver sessions suitable for specific health conditions.

## Figures and Tables

**Table 1:** Socio-demographic characteristics of the yoga providers.

	All	Yoga Teacher	Yoga Therapist	
	*n* (%)	*n* (%)	*n* (%)	*p*
Gender				
Female	379 (93.12)	313 (93.43)	62 (92.54)	0.19
Male	23 (5.65)	19 (5.67)	3 (4.48)	
Other	1 (0.25)	1 (0.30)	0	
Prefer not to say	3 (0.74)	1 (0.30)	2 (2.99)	
Missing	1 (0.25)	1 (0.30)	0	
Age (years)				
18–24	0	0	0	0.12
25–44	60 (14.74)	51 (15.22)	7 (10.45)	
45–64	280 (68.80)	231 (68.96)	46 (68.66)	
65+	64 (15.72)	52 (15.52)	12 (17.91)	
Prefer not to say	3 (0.74)	1 (0.30)	2 (2.99)	
Ethnic group				
White	378 (92.87)	314 (93.73)	59 (88.06)	0.29
Asian/Asian British	6 (1.47)	4 (1.19)	2 (2.99)	
Black/African/Caribbean/Black British	1 (0.25)	1 (0.30)	0	
Mixed/Multiple ethnic groups	8 (1.97)	6 (1.79)	2 (2.99)	
Other	6 (1.47)	5 (1.49)	1 (1.49)	
Prefer not to say	8 (1.97)	5 (1.49)	3 (4.48)	
Yoga education level				
Certificate	121 (29.73)	106 (31.74)	12 (28.36)	<0.001
Diploma	199 (48.89)	172 (51.50)	26 (38.81)	
Bachelor	18 (4.42)	14 (4.19)	4 (5.97)	
Higher education	52 (12.78)	32 (9.58)	19 (28.36)	
Other	16 (3.93)	10 (2.99)	6 (8.96)	
Missing	1 (0.25)	1 (0.29)	0	
Yoga associations *				
British Wheel of Yoga	252 (54.90)	211 (57.49)	39 (44.82)	0.45
Iyengar Yoga UK	108 (23.52)	86 (23.43)	20 (22.98)	0.47
Independent Yoga Network	27 (5.88)	23 (6.26)	4 (4.59)	0.78
Yoga Alliance Professionals	20 (4.35)	16 (4.35)	4 (4.59)	0.75
Yoga Alliance Internationals	15 (3.26)	10 (2.72)	5 (5.74)	0.07
Other	31 (6.75)	16 (4.35)	14 (16.09)	<0.001
Missing	6 (1.30)	5 (1.36)	1 (1.14)	
Current yoga teaching role *				
Self-employed	369 (82.00)	299 (81.47)	66 (84.62)	0.01
Employed (public sector)	44 (9.77)	36 (9.80)	8 (10.26)	1.00
Employed (private sector)	19 (4.22)	15 (4.08)	3 (3.85)	0.78
Other	17 (3.77)	16 (4.35)	1 (1.28)	0.32
Missing	1 (0.22)	1 (0.27)	0	
A member of the statutorily regulated healthcare profession				
Yes	32 (7.86)	28 (8.36)	4 (5.97)	0.90
No	373 (91.65)	305 (91.04)	63 (94.03)	
Missing	2 (0.49)	2 (0.60)	0	
Teaching experience (in years)				
Median	10	10	15	<0.001
Min.	0.5	0.5	2	
Max.	50	50	50	

* Participants were allowed to choose more than one response.

**Table 2:** Structure, delivery, and cost of yoga sessions.

	All	Yoga Teacher	Yoga Therapist	
	*n* (%)	*n* (%)	*n* (%)	*p*
Delivery of group yoga sessions				
Yes	387 (95.09)	319 (95.22)	63 (94.03)	1.00
No	19 (4.67)	16 (4.78)	3 (4.48)	
Missing	1 (0.25)	0	1 (1.49)	
Delivery of one-to-one yoga sessions				
Yes	127 (31.20)	82 (24.48)	44 (65.67)	<0.001
No	277 (68.06)	250 (74.63)	23 (34.33)	
Missing	3 (0.74)	3 (0.90)	0	
Evening yoga sessions				
Yes	326 (80.10)	267 (79.70)	55 (82.09)	0.55
No	78 (19.16)	66 (19.70)	11 (16.42)	
Missing	3 (0.74)	2 (0.60)	1 (1.49)	
Weekend yoga sessions				
Yes	104 (25.55)	73 (21.79)	27 (40.30)	<0.001
No	300 (73.71)	260 (77.61)	39 (58.21)	
Missing	3 (0.74)	2 (0.60)	1 (1.49)	
Proportion of female attendees				
0–25%	2 (0.49)	2 (0.60)	0	0.16
25–50%	11 (2.70)	10 (2.99)	1 (1.49)	
50–75%	133 (32.68)	103 (30.75)	30 (44.78)	
75–100%	257 (63.14)	216 (64.48)	36 (53.73)	
Missing	4 (0.98)	4 (1.19)	0	
Payment method of yoga sessions *				
Out-of-pocket payment by yoga attendees	381 (81.75)	312 (81.03)	65 (85.52)	0.22
Public funded (e.g., NHS)	28 (6.00)	22 (5.71)	5 (6.57)	0.78
Private insurance	0	0	0	
Other (e.g., charity-funded)	52 (11.15)	47 (12.20)	5 (6.57)	0.14
Missing	5 (1.07)	4 (1.03)	1 (1.31)	
Setting of yoga sessions *				
In private (non-government) settings	321 (60)	257 (58.54)	59 (67.04)	0.03
In public (government) settings	71 (13.27)	61 (13.89)	8 (9.09)	0.21
In public and private partnership settings	97 (18.13)	82 (18.67)	15 (17.04)	0.72
Other (e.g., online, outdoors)	40 (7.47)	34 (7.74)	5 (5.68)	0.50
Missing	6 (1.12)	5 (1.13)	1 (1.13)	
Regions				
Wales	6 (1.47)	3 (0.90)	3 (4.48)	0.06
Scotland	28 (6.87)	23 (6.87)	5 (7.46)	0.88
Northern Ireland	4 (0.98)	4 (1.19)	0	1.00
North East	11 (2.70)	10 (2.99)	1 (1.49)	0.48
North West	48 (11.79)	42 (12.54)	6 (8.96)	0.38
Yorkshire and the Humber	39 (9.58)	35 (10.45)	4 (5.97)	0.36
East Midlands	39 (9.58)	33 (9.85)	6 (8.96)	0.79
West Midlands	20 (4.91)	19 (5.67)	1 (1.49)	0.22
East	36 (8.84)	26 (7.76)	10 (14.93)	0.06
London	53 (13.02)	35 (10.45)	18 (26.87)	<0.001
South East	54 (13.26)	45 (13.43)	9 (13.43)	0.96
South West	55 (13.51)	48 (14.33)	7 (10.45)	0.37
Missing	17 (4.17)	15 (4.48)	2 (2.95)	
	Median (IQR)	Median (IQR)	Median (IQR)	*p*
Number of group yoga sessions per week	4 (2, 6)	3 (2, 6)	6 (3, 8)	<0.001
Number of attendees in a group yoga session	10 (8, 15)	10 (8, 15)	12 (9, 15)	0.01
Duration of a group yoga session (min)	90 (67.5, 90)	90 (67.5, 90)	90 (65, 90)	0.56
Cost of a group yoga session (GBP)	7 (6, 8.75)	7 (6, 8)	8 (6, 10)	<0.001
Number of one-to-one yoga sessions per week	2 (1, 3)	2 (1, 3)	3 (2, 4)	<0.001
Duration of a one-to-one yoga session (min)	60 (60, 75)	60 (60, 75)	60 (60, 75)	0.27
Cost of a one-to-one yoga session (GBP)	40 (30, 50)	35 (30, 45)	40 (30, 50)	<0.001

* Participants were allowed to choose more than one response.

**Table 3:** Style and content of yoga sessions.

	All	Yoga Teacher	Yoga Therapist	
	*n* (%)	*n* (%)	*n* (%)	*p*
Style of yoga *				
Iyengar	162 (26.04)	131 (25.63)	29 (27.88)	0.54
Asthanga	32 (5.14)	24 (4.69)	7 (6.73)	0.36
Vinyasa	91 (14.63)	78 (15.26)	12 (11.54)	0.32
Bikram	2 (0.32)	1 (0.19)	1 (0.96)	0.30
Kundalini	9 (1.44)	8 (1.56)	1 (0.96)	1.00
Yin	58 (9.32)	46 (9.00)	11 (10.58)	0.57
Viniyoga	37 (5.94)	24 (4.69)	13 (12.50)	<0.001
Hatha	172 (27.65)	152 (29.73)	18 (17.30)	<0.001
Other	56 (9.00)	44 (8.60)	12 (11.53)	
Missing	3 (0.48)	3 (0.58)	0	
Number of yoga styles used				
1 style	277 (68.06)	228 (68.06)	45 (67.16)	0.96
2 styles	68 (16.71)	56 (16.72)	12 (17.91)	
3 or more styles	59 (13.95)	48 (14.32)	10 (14.93)	
Missing	3 (0.74)	3 (0.90)	0	
Yama (rules of moral code) **				
0–25% (i.e., none or little emphasis)	178 (43.73)	157 (46.87)	20 (29.85)	0.01
25–50% (i.e., some emphasis)	134 (32.92)	104 (31.04)	27 (40.30)	
50–75% (i.e., a good deal of emphasis)	26 (6.39)	18 (5.37)	8 (11.94)	
75–100% (i.e., a great deal of emphasis)	19 (4.67)	12 (3.58)	6 (8.96)	
Missing	50 (12.29)	44 (13.13)	6 (8.96)	
Niyama (rules of personal behavior) **				
0–25% (i.e., none or little emphasis)	171 (42.01)	150 (44.78)	20 (29.85)	<0.001
25–50% (i.e., some emphasis)	144 (35.38)	116 (34.63)	25 (37.31)	
50–75% (i.e., a good deal of emphasis)	28 (6.88)	19 (5.67)	8 (11.94)	
75–100% (i.e., a great deal of emphasis)	17 (4.18)	10 (2.99)	7 (10.45)	
Missing	47 (11.55)	40 (11.94)	7 (10.45)	
Asana (yogic poses) **				
0–25% (i.e., none or little emphasis)	4 (0.98)	3 (0.90)	1 (1.49)	0.07
25–50% (i.e., some emphasis)	15 (3.69)	10 (2.99)	5 (33.33)	
50–75% (i.e., a good deal of emphasis)	140 (34.40)	110 (32.84)	28 (41.79)	
75–100% (i.e., a great deal of emphasis)	242 (59.46)	206 (61.49)	33 (49.25)	
Missing	6 (1.47)	6 (1.79)	0	
Pranayama (breathing practices) **				
0–25% (i.e., none or little emphasis)	60 (14.74)	51 (15.22)	9 (13.43)	0.03
25–50% (i.e., some emphasis)	154 (37.84)	134 (40.00)	16 (23.88)	
50–75% (i.e., a good deal of emphasis)	116 (28.50)	90 (26.87)	26 (38.81)	
75–100% (i.e., a great deal of emphasis)	72 (17.69)	55 (16.42)	16 (23.88)	
Missing	5 (1.23)	5 (1.49)	0	
Pratyahara (withdrawal of senses) **				
0–25% (i.e., none or little emphasis)	150 (36.86)	135 (40.30)	14 (20.90)	<0.001
25–50% (i.e., some emphasis)	119 (29.24)	97 (28.96)	19 (28.36)	
50–75% (i.e., a good deal of emphasis)	55 (13.51)	42 (12.54)	13 (19.40)	
75–100% (i.e., a great deal of emphasis)	21 (5.16)	9 (2.69)	11 (16.42)	
Missing	62 (15.23)	52 (15.52)	10 (14.93)	
Dharana (concentration) **				
0–25% (i.e., none or little emphasis)	108 (26.54)	92 (27.46)	15 (22.39)	<0.001
25–50% (i.e., some emphasis)	129 (31.70)	108 (32.24)	19 (28.36)	
50–75% (i.e., a good deal of emphasis)	81 (19.90)	68 (20.30)	12 (17.91)	
75–100% (i.e., a great deal of emphasis)	39 (9.58)	23 (6.87)	15 (22.39)	
Missing	50 (12.29)	44 (13.13)	6 (8.96)	
Dhyana (meditation) and relaxation practices **				
0–25% (i.e., none or little emphasis)	80 (19.66)	68 (20.30)	11 (16.42)	0.28
25–50% (i.e., some emphasis)	159 (39.07)	132 (39.40)	24 (35.82)	
50–75% (i.e., a good deal of emphasis)	92 (22.62)	74 (22.09)	17 (25.37)	
75–100% (i.e., a great deal of emphasis)	50 (12.29)	37 (11.04)	13 (19.40)	
Missing	26 (6.39)	24 (7.16)	2 (2.99)	
Samadhi (transcendence) **				
0–25% (i.e., none or little emphasis)	260 (63.88)	219 (65.37)	38 (56.72)	<0.001
25–50% (i.e., some emphasis)	36 (8.85)	27 (8.06)	7 (10.45)	
50–75% (i.e., a good deal of emphasis)	15 (3.69)	7 (2.09)	8 (11.94)	
75–100% (i.e., a great deal of emphasis)	4 (0.98)	2 (0.60)	2 (2.99)	
Missing	92 (22.60)	80 (23.88)	12 (17.91)	

* Participants were allowed to choose more than one response. ** Participants were told that some of these may not feature at all in yoga sessions (e.g., Samadhi) and may be covered throughout the session (e.g., pranayama) so the total does not need to add up to 100%.

**Table 4:** Yoga providers’ awareness of health conditions and their training on hypertension.

	All	Yoga Teacher	Yoga Therapist	
	*n* (%)	*n* (%)	*n* (%)	*p*
Disclosure of health conditions by yoga attendees				
Never	4 (0.98)	4 (1.19)	0	0.40
Rarely/Sometimes	101 (24.82)	86 (25.67)	13 (19.40)	
Often	299 (73.46)	242 (72.24)	54 (80.60)	
Missing	3 (0.74)	3 (0.90)	0	
Disclosure of hypertension/high blood pressure by yoga attendees				
Never	34 (8.35)	28 (8.36)	5 (7.46)	0.14
Rarely/Sometimes	242 (59.46)	205 (61.20)	35 (51.24)	
Often	103 (25.31)	78 (23.28)	24 (35.82)	
Missing	28 (6.88)	24 (7.16)	3 (4.48)	
Disclosure of diabetes by yoga attendees				
Never	99 (24.32)	86 (25.67)	12 (17.91)	0.11
Rarely/Sometimes	230 (56.51)	183 (54.63)	43 (64.18)	
Often	24 (5.90)	17 (5.07)	7 (10.45)	
Missing	54 (13.27)	49 (14.63)	5 (7.46)	
Disclosure of obesity by yoga attendees				
Never	156 (38.33)	133 (39.70)	22 (32.84)	0.29
Rarely/Sometimes	164 (40.29)	128 (38.21)	33 (49.25)	
Often	17 (4.18)	14 (4.18)	2 (2.98)	
Missing	70 (17.20)	60 (17.91)	10 (14.93)	
Disclosure of heart diseases (e.g., coronary heart disease, angina, history of myocardial infarction, etc.) by yoga attendees				
Never	91 (22.36)	81 (24.18)	9 (13.43)	0.06
Rarely/Sometimes	232 (57.00)	188 (56.12)	40 (59.70)	
Often	38 (9.34)	28 (8.36)	10 (14.93)	
Missing	46 (11.30)	38 (11.34)	8 (11.94)	
Disclosure of stroke by yoga attendees				
Never	205 (50.37)	174 (51.94)	28 (41.79)	0.12
Rarely/Sometimes	126 (30.96)	97 (28.95)	27 (40.30)	
Often	11 (2.70)	8 (2.39)	3 (4.48)	
Missing	65 (15.97)	56 (16.72)	9 (13.43)	
Disclosure of chronic kidney disease by yoga attendees				
Never	254 (62.41)	211 (62.99)	39 (58.21)	0.21
Rarely/Sometimes	82 (20.15)	64 (19.10)	17 (25.37)	
Often	5 (1.23)	3 (0.90)	2 (2.99)	
Missing	66 (16.21)	57 (17.01)	9 (13.43)	
Disclosure of respiratory diseases (e.g., pulmonary hypertension) by yoga attendees				
Never	103 (25.31)	91 (27.16)	11 (16.42)	0.04
Rarely/Sometimes	226 (55.53)	182 (54.33)	40 (59.70)	
Often	27 (6.63)	19 (5.67)	8 (11.94)	
Missing	51 (12.53)	43 (12.84)	8 (11.94)	
Disclosure of mental issues (e.g., depression, stress, anxiety) by yoga attendees				
Never	21 (5.16)	19 (5.67)	2 (2.99)	0.31
Rarely/Sometimes	201 (49.38)	168 (50.15)	31 (46.26)	
Often	166 (40.79)	130 (38.81)	33 (49.25)	
Missing	19 (4.67)	18 (5.37)	1 (1.5)	

## Data Availability

A de-identified data set will be available upon request unless there are legal or ethical reasons for not doing so.
